# Speechreading Ability Is Related to Phonological Awareness and Single-Word Reading in Both Deaf and Hearing Children

**DOI:** 10.1044/2020_JSLHR-20-00159

**Published:** 2020-10-27

**Authors:** Elizabeth Buchanan-Worster, Mairéad MacSweeney, Hannah Pimperton, Fiona Kyle, Margaret Harris, Indie Beedie, Amelia Ralph-Lewis, Charles Hulme

**Affiliations:** a Institute of Cognitive Neuroscience, University College London, United Kingdom; b Deafness Cognition and Language Research Centre, University College London, United Kingdom; c Faculty of Health and Life Sciences, Oxford Brookes University, United Kingdom; d Department of Education, University of Oxford, United Kingdom

## Abstract

**Purpose:**

Speechreading (lipreading) is a correlate of reading ability in both deaf and hearing children. We investigated whether the relationship between speechreading and single-word reading is mediated by phonological awareness in deaf and hearing children.

**Method:**

In two separate studies, 66 deaf children and 138 hearing children, aged 5–8 years old, were assessed on measures of speechreading, phonological awareness, and single-word reading. We assessed the concurrent relationships between latent variables measuring speechreading, phonological awareness, and single-word reading.

**Results:**

In both deaf and hearing children, there was a strong relationship between speechreading and single-word reading, which was fully mediated by phonological awareness.

**Conclusions:**

These results are consistent with ideas from previous studies that visual speech information contributes to the development of phonological representations in both deaf and hearing children, which, in turn, support learning to read. Future longitudinal and training studies are required to establish whether these relationships reflect causal effects.

Deaf children and adults tend to have poorer reading skills, on average, than their hearing peers (e.g., Conrad, 1979; Qi & Mitchell, 2011). A U.K. study from 40 years ago (Conrad, 1979) found that deaf school leavers aged 16 years had average reading levels equivalent to a 9-year-old. Despite subsequent advances in amplification technology, the gap in reading attainment between deaf and hearing children has remained large (Harris et al., 2017b).

Phonological awareness is the ability to represent and manipulate the sublexical structure of spoken words. Phonological awareness skills are strongly related to single-word reading skills in hearing children (Hulme & Snowling, 2012; Melby-Lervåg et al., 2012). Deaf children tend to have poorer phonological awareness than their hearing peers, as measured by both phoneme- and rhyme-matching tasks (Dyer et al., 2003; Johnson & Goswami, 2010; Kyle & Harris, 2006), but the extent to which phonological skills are important for reading development in deaf children has been the focus of extensive debate. Some studies have found a moderate positive correlation between performance on phonological awareness tasks and both single-word and passage reading (rhyme judgment *r* = .39, pseudohomophones *r* = .46, Dyer et al., 2003; *r* = .43, Harris & Beech, 1998; *r* = .71, Herman, Kyle, & Roy, 2019; *r* = .74, Colin et al., 2007) but several others have not (e.g., Hanson & Fowler, 1987; Kyle & Harris, 2006; Leybaert & Alegria, 2003; Mayberry et al., 2011). A meta-analysis found that, on average, phonological awareness explained only 11% of the variance in deaf individual's reading scores (Mayberry et al., 2011). However, this is similar to findings in hearing children (12%; Bus & Van Ijzendoorn, 1999). It should also be noted that the Mayberry et al. (2011) meta-analysis included studies of both deaf children and adults, but phonological awareness skills are known to be particularly closely related to reading skill among beginner readers.

The development of phonological representations of spoken words is heavily dependent upon auditory speech information but almost certainly depends upon other sources of information as well for both deaf and hearing children, including visual speech information (Jerger et al., 2009), and a person's own articulation (Carroll et al., 2003; Johnson & Goswami, 2010). Because deaf children have reduced access to auditory information, they are likely to place a greater reliance on nonauditory information when developing phonological representations of speech. In support of this proposal, deaf children who use Cued Speech, a system that uses manual gestures to disambiguate speechread phonetic information (Cornett, 1967), generally have better phonological awareness and reading skills than deaf children who do not (e.g., Colin et al., 2007).

Visual and auditory speech information are complementary, because some phonemes that are hard to distinguish by sound are easy to distinguish visually (e.g., /n/ and /m/) and vice versa (e.g., /m/ and /b/; Campbell, 2011). In the current article, we use visual speech to refer to visually presented speech information with or without accompanying auditory speech. When visual speech is congruent with auditory speech, it can enhance speech perception in hearing adults and children (Knowland et al., 2016; Lusk & Mitchel, 2016; Sumby & Pollack, 1954), while incongruent visual information can disrupt it. For example, a visually presented /ga/ that is synchronous with an aurally presented /ba/ is perceived as a /da/ (McGurk & MacDonald, 1976).

Given the role of visual information in audiovisual speech perception, it is likely that visual information contributes to the development of phonological representations in hearing children even though they have full access to auditory speech information. Infants as young as 2 months old are sensitive to the correspondence between auditory and visual speech information (Patterson & Werker, 2003), and visual information has been shown to influence phonetic discrimination (Jerger et al., 2018; Teinonen et al., 2008; Weikum et al., 2007), which in turn predicts spoken language development (Kuhl et al., 2005). Visual speech perception also influences word form recognition (Weatherhead & White, 2017) and vocabulary development in hearing infants and children (Altvater-Mackensen & Grossmann, 2015; Erdener & Burnham, 2018; Jerger et al., 2018). In children with developmental language disorder, Heikkilä et al. (2018) found that audiovisual speech perception training led to improvements on a nonword repetition task (used as a measure of phonological representations) in contrast to auditory-only training. These findings support the idea that visual information contributes to the development of phonological representations in hearing children.

Speechreading (lipreading) refers to visual speech perception in the absence of auditory information. Speechreading skill is highly variable in both deaf and hearing populations (Bernstein et al., 2000; Kyle et al., 2013). Studies with hearing adults suggest that variability in speechreading skill is related to a number of different factors, including the individual's vocabulary skills (Lyxell & Holmberg, 2000), verbal short-term memory (Lyxell & Ronnberg, 1993), reading level (Mohammed et al., 2006), age (Tye-Murray et al., 2007), and experience with speechreading (Bernstein et al., 2000). For both deaf and hearing children, speechreading ability improves with age, and speechreading single words is easier than sentences or stories (Dodd et al., 1998; Green et al., 1981; Kyle et al., 2013; Kyle & Harris, 2010). Deaf children who use spoken language as their preferred communication tend to be better at speechreading, as do those with higher levels of residual hearing (Kyle et al., 2016).

In deaf children and adults, speechreading is correlated with performance on explicit phonological awareness tasks, such as rhyme judgments (Campbell & Wright, 1988), both concurrently (*r* = .46; Kyle & Harris, 2006; Mohammed et al., 2006) and longitudinally (Kyle & Harris, 2010; *r* = .43, Harris et al., 2017a). Deaf adolescents show recency effects in speechread serial recall tasks (Dodd et al., 1983) and use speechread information in rhyme-matching tasks (Dodd & Hermelin, 1977). Spelling errors among orally educated deaf children also reflect confusions that are common in speechreading (e.g., Leybaert & Alegria, 1995; Sutcliffe et al., 1999). These findings suggest that the information conveyed by visual speech perception is important for the development of phonological representations in deaf children. Numerous studies show a positive relationship between speechreading and reading proficiency (accuracy and comprehension) in deaf children, concurrently (Arnold & Köpsel, 1996; *r* = .49, Kyle et al., 2016; *r* = .60, Kyle & Harris, 2006) and longitudinally (*r* = .53, Harris et al., 2017a; *r* = .64, Kyle & Harris, 2010) regardless of whether a child uses spoken or signed language (Kyle & Harris, 2010, 2011). Perhaps surprisingly, speechreading also relates to reading (accuracy of passage reading *r* = .31, comprehension *r* = .28, Kyle et al., 2016; single-word reading *r* = .58, Kyle & Harris, 2011) and phonological awareness (rhyme and alliteration judgment *r* = .59; Kyle & Harris, 2011) in hearing children. Furthermore, speechreading is a predictor of reading ability independently of vocabulary skills in both deaf (8% variance explained) and hearing children (2% variance explained; Kyle et al., 2016). Several researchers have suggested that speechreading contributes to the development of phonological representations and thereby facilitates reading development (Kyle, 2015; Leybaert, 1993; Mohammed et al., 2006).

The evidence reviewed above strongly suggests that speechreading may contribute to the development of phonological representations in young deaf children, which in turn may lead to better single-word reading ability. In Study 1, we investigate whether the relationship between speechreading and single-word reading is mediated by phonological awareness in a concurrent study with 5- to-8-year-old deaf children. In Study 2, we asked the same question about the relationship between these variables in hearing children.

## Study 1: Deaf Children

## Method

### Participants

The study was granted ethical approval by University College London's Research Ethics Committee. The data in the current study were collected at the first time point of a speechreading training study (Pimperton et al., 2019), prior to any training. Informed parental consent was provided for 66 severely to profoundly deaf children (35 boys, 31 girls) from specialist and mainstream schools from across England. All children had a bilateral hearing loss. For almost all children, onset of deafness was before 12 months. One child had onset of deafness at 48 months. The children had a range of demographic, audiological, and communicative characteristics, representative of the population of deaf children. These characteristics are summarized in Table 1.

**Table 1.** Participant characteristics for the deaf children.

Characteristic	
Age, *M* (*SD*) range	6 years (7.8 months) 59–94 months
Unaided category of deafness^a^	
Severe	25
Profound	41
Device use	
CI bilaterally	29
HA bilaterally	31
One HA, one CI	2
No device	4
Classroom communication	
BSL only	13
Mixture of speech and sign^b^	33
Spoken English only	20

*Note.* CI = Cochlear implant; HA = hearing aid; BSL = British Sign Language.

a Five children had a moderate hearing loss in their better ear, but severe or profound in their contralateral ear, and so were included in this study in the severe category.

b The extent to which children used either speech or sign in the classroom varied.

### Measures

All tasks were administered to children individually in a quiet space in their school. Instructions were provided in the child's preferred language (English or British Sign Language [BSL]).

#### Speechreading

*Test of Child Speechreading Everyday Questions extension.* Speechreading ability was assessed using the Test of Child Speechreading (TOCS) Everyday Questions extension task (Kyle et al., 2013; https://dcalportal.org/tests). Children watched 12 silent videos of a male or female model asking everyday questions, such as “How old are you?” The child's task was to repeat, in English or BSL, as much of the question as they could. Children were awarded 1 point for each lexical item in the sentence correctly repeated regardless of the word order (maximum score = 62).

#### Reading

*York Assessment of Reading Comprehension.* Single-word reading was assessed using the York Assessment of Reading Comprehension (YARC) Early Word and Single-Word Reading subtests (Hulme et al., 2009). Children could respond in either BSL or English. The YARC Reading subtests consist of lists of regular and irregular single words (30 and 60 words, respectively).

*Word–picture matching.* A word–picture matching task (Pimperton et al., 2019) was used to measure single-word reading independently of speech production proficiency. The child was asked to point to one of four pictures in each corner of the screen to indicate what the word in the center of the screen meant. On each of the 24 trials, the target picture (e.g., “bat”) was presented, along with two phonological distractors (e.g., “bun,” “hat”) and an unrelated picture (e.g., “ring”). The word–picture matching task had a maximum score of 24.

*Phonological awareness.* Word onset and rime awareness were tested in a novel task from Pimperton et al. (2019). On each of the 24 trials, a target picture was placed at the top of the screen, and each child was asked to point to which of the three pictures at the bottom of the screen started/ended with the same sound as the target. This task was used to assess phonological awareness skills without requiring a verbal response, which would confound phonological awareness skills and speech production skills.

On each trial, there was the target (e.g., “bees”); the matching image (e.g., “peas”); a near distractor (e.g., “cars”), which shared some phonology and orthography with the target; and a far distractor (e.g., “cave”) that was not similar phonologically or orthographically to the target. The task was split into two sections, with the first half assessing alliterative matches (e.g., “dog” and “duck”) and the second half assessing rhyming matches (e.g., “bees” and “peas”). Before the beginning of each section, the task was explained to the child, and they were given two practice trials. They were given help if needed on the practice trials by saying the target word paired with each of the distractor types (e.g., “bees-peas, bees-cars, bees-cave”). When they got the correct answer, they were given positive feedback, and the matching element of the words was emphasized (e.g., “That's right, ‘dog’ and ‘duck’ because they both start with d-d-d”). After the two practice trials, there were 12 test trials for which they were not given any feedback. The word onset and rime awareness task had a maximum score of 24 (12 points in each half).

## Results

Descriptive statistics for all measures are shown in Table 2, and the correlations between measures are shown in Table 3. The mean standard score on the YARC (Hulme et al., 2009) Single-Word Reading subtest was 82.47 (*SD* = 18.50, range: 70–130), indicating that this sample was of below average reading ability, as expected. It is important to note, however, that the sample shows a very wide range of single-word reading scores.

**Table 2.** Means (standard deviations) and reliabilities for the measures of speechreading, reading, and phonological awareness in deaf children.

Task	*M* (*SD*)	Range	Cronbach's α
Speechreading^a^ (max = 62)	5.86 (10.63)	0–52	.94
YARC EW (max = 30)	11.00 (10.54)	0–30	.98
YARC SW (max = 60)	7.02 (10.15)	0–43	.97
Word–picture matching (max = 24)	11.76 (6.90)	1–24	.92
PA onset matching score (max = 12)	5.32 (2.89)	0–12	.72
PA rime matching score (max = 12)	5.18 (2.69)	1–12	.70

*Note.* YARC = York Assessment of Reading Comprehension; EW = Early Word Reading subtest; SW = Single-Word Reading subtest; PA = phonological awareness.

a Test of Child Speechreading.

**Table 3.** Correlations between measures of speechreading, phonological awareness, and reading for deaf children.

Task	Speechreading	YARC EW	YARC SW	Word–picture matching	Onset score	Rime score
Speechreading^a^	—	.579	.631	.540	.451	.587
YARC EW		—	.878	.923	.539	.502
YARC SW			—	.841	.632	.611
Word–picture matching				—	.596	.529
PA onset matching score					—	.506

*Note.* All measures were highly correlated with each other (*p* < .001). YARC = York Assessment of Reading Comprehension; EW = Early Word Reading subtest; SW = Single-Word Reading subtest; PA = phonological awareness.

a Test of Child Speechreading.

Our primary aim was to assess whether the relationship between speechreading and single-word reading is mediated by phonological awareness. The analyses were conducted as a series of path models in Mplus (Version 8; Muthén & Muthén, 2017), using robust maximum likelihood estimators to account for some measures not being normally distributed. Missing values were handled using full-information maximum likelihood estimators (default in Mplus). To assess the relationship between speechreading, phonological awareness, and single-word reading, we constructed a latent variable model with three constructs: Speechreading, Phonological Awareness, and Reading. A latent variable for speechreading was estimated by item parceling, using scores from every third sentence from the TOCS Everyday Questions to form three observed variables. A latent variable for single-word reading was defined by scores from the YARC Single-Word and Early Word Reading subtests and the word–picture matching task. A latent variable for phonological awareness was defined by scores from the onset- and rime-matching tasks. The sample differed greatly in age, but analyses showed that age was not significantly related to any of the measures in the model, so age was not considered further in the analyses.

**Figure 1.**
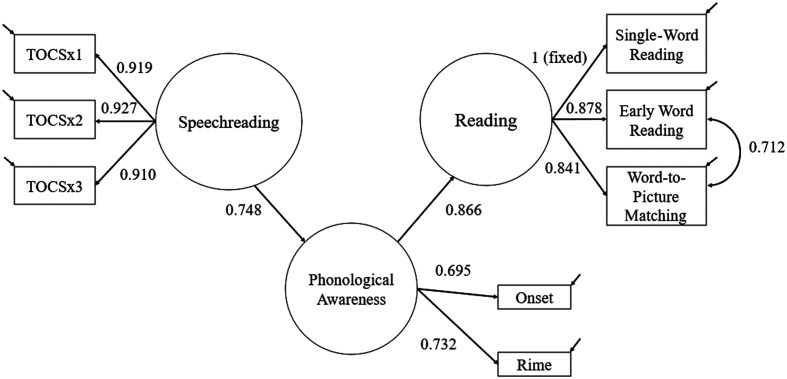
Path model showing the indirect relationship between speechreading and single-word reading in deaf children. Standardized regression coefficients are shown. The Test of Child Speechreading (TOCS) was parceled into three observed variables (TOCSx1, TOCSx2, and TOCSx3), which each contained a third of the test items.

We used an iterative approach to construct the final model shown in Figure 1. In the first stage, Speechreading was regressed onto Reading. There was a strong positive relationship between Speechreading and Reading (β = 0.659). In the second stage, the Phonological Awareness latent variable was added as a mediating factor between Speechreading and Reading. In the initial model, the residual variance for the YARC Single-Word Reading subtest was small but negative and so was fixed to zero.

The final model showed full mediation, with the effect from Speechreading to Reading being entirely accounted for by the indirect pathway: Speechreading–Phonological Awareness–Reading. Dropping the nonsignificant direct path between Speechreading and Reading did not result in any appreciable loss of fit for the model fit (χ^2^ difference = 0.006, *df* = 1, *p* > .10). The indirect effect, via Phonological Awareness, was statistically reliable using bootstrapped standard errors (0.648, 95% CI [0.361, 0.860]). Overall, this model provided an excellent fit to the data, χ^2^(18, *n* = 66) = 23.302, *p* = .179, comparative fit index = 0.990, Tucker–Lewis index = 0.984, RMSEA = 0.067, 95% CI [0.000, 0.136], SRMR = 0.034, and accounts for 75% of the variance in single-word reading ability.

## Discussion

The concurrent relationship between speechreading and single-word reading ability in young deaf children is mediated by phonological awareness. This finding is consistent with the idea that speechreading contributes to the development of phonological representations in deaf children and, in turn, plays a role in reading development (Kyle, 2015). In Study 2, we explore whether the same is true in hearing children. Some of the tests used with deaf children in Study 1 were changed to be more appropriate for use with hearing children.

## Study 2: Hearing Children

## Method

### Participants

The study was granted ethical approval by University College London's Research Ethics Committee. Informed parental consent was obtained for 138 hearing children of ages 5–8 years (79 boys, 59 boys) from two schools in Cambridgeshire and one school in London. Two children were excluded, because one did not speak English and the second withdrew. Characteristics of the remaining hearing children are shown in Table 4. There were 13 children who had not learned English from birth. They had been learning English for an average of 3 years (*SD* = 1.2 years, range: 1–5 years), as reported by their parents. These 13 children had average scores on the British Ability Scales Word Definitions subtest (average *T* score = 51, range: 38–64) and so were included in the study.

**Table 4.** Participant characteristics for the hearing children.

Characteristic	
Age, *M (SD)* range	6 years 5 months (8.5 months) 62–98 months
Home language	
English	91
English + Other	32
Other (not English)	13

### Measures

The same measures of speechreading and single-word reading were used as in Study 1, except for the word–picture matching task, which was not administered to the hearing children as ceiling effects were anticipated for this group on this task. Large differences in levels of phonological ability between our deaf and hearing samples necessitated the use of a different measure of phonological ability to that used in Study 1 (which would have been too easy for the children in our hearing sample). We used the YARC Sound Deletion subtest (Hulme et al., 2009) as our measure of phonological awareness here because this task assesses phonemic awareness, which is known to be particularly closely related to single-word reading ability (Hulme, 2002; Hulme et al., 1998). This task has good measurement sensitivity and has been shown to be highly predictive of reading ability in hearing children in the age range studied here (e.g., Hulme et al., 2012; Thompson et al., 2015).

## Results

Descriptive statistics for all measures are shown in Table 5 and the correlations between measures in Table 6. The mean standard score on the YARC (Hulme et al., 2009) Single-Word Reading subtest was 108.85 (*SD* = 16.80, range: 70–130), indicating that this sample was of slightly above average reading ability, but with a very wide range of scores.

**Table 5.** Means (standard deviations) and reliabilities for the measures of speechreading, reading, spelling, and phoneme deletion for hearing children.

Task	*M* (*SD*)	Range	Cronbach's α
Speechreading^a^ (max = 62)	16.95 (10.10)	0–47	.79
YARC EW (max = 30)	24.63 (7.56)	0–30	.96
YARC SW (max = 60)	25.92 (10.69)	0–56	.97
Phoneme deletion (max = 12)	7.96 (2.75)	0–12	.79

*Note.* YARC = York Assessment of Reading Comprehension; EW = Early Word Reading subtest; SW = Single-Word Reading subtest.

a Test of Child Speechreading.

**Table 6.** Correlations between measures of speechreading, phonological awareness and reading for hearing children.

Task	Speechreading	YARC EW	YARC SW	Phoneme deletion
Speechreading^a^	—	.494	.564	.471
YARC EW		—	.814	.771
YARC SW			—	.759

*Note.* All correlations were significant (*p* < .001). YARC = York Assessment of Reading Comprehension; EW = Early Word Reading subtest; SW = Single-Word Reading subtest.

a Test of Child Speechreading.

As in Study 1, our primary aim was to assess whether the relationship between speechreading and single-word reading is mediated by phonological awareness. We assessed this by constructing an equivalent path model to that used in Study 1, with three latent variables: Speechreading, Phoneme Awareness, and Reading. A latent variable for speechreading was estimated by item parceling, using scores from every third sentence from the TOCS Everyday Questions to form three observed variables. A latent variable for single-word reading was estimated by performance on the YARC Single-Word and Early Word Reading subtests. A latent variable for phoneme awareness was defined by a single indicator with measurement error estimated as 1 − reliability of the measure. Because of the very wide age range in the sample, all latent variables were regressed on age (for simplicity, those regressions are not show in the path diagram).

**Figure 2.**
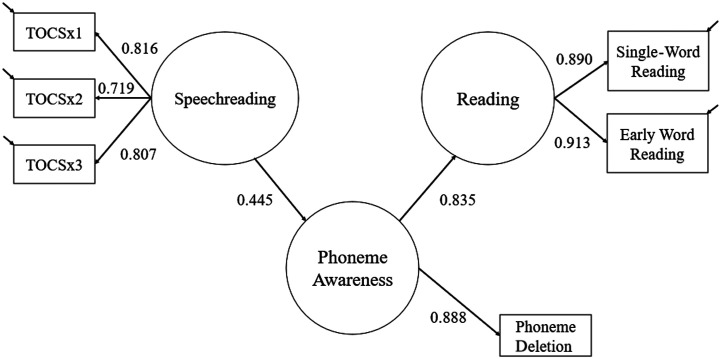
Path model showing the indirect relationship between speechreading and single-word reading in hearing children. Standardized regression coefficients are shown. The Test of Child Speechreading (TOCS) was parceled into three observed variables (TOCSx1, TOCSx2, and TOCSx3), which each contained a third of the test items. Because of the very wide age range in the sample, all latent variables were regressed on age. In addition, the single-word reading task was correlated with TOCSx1 and TOCSx2. For clarity of presentation, these regressions are not shown, but the coefficients reflect their inclusion in the model.

The model (see Figure 2) showed full mediation, with the effect from Speechreading to Reading (β = 0.369) being entirely accounted for by the indirect pathway (Speechreading–Phoneme Awareness–Reading). The indirect effect, via phoneme awareness, was statistically reliable as assessed by bootstrapped standard errors (standardized indirect effect = 0.372, 95% CI [0.251, 0.493]). Dropping the nonsignificant direct path between Speechreading and Reading resulted in no appreciable loss of model fit (χ^2^ difference = 0.002, *p* > .10). Overall, this model provided an excellent fit to the data, χ^2^(9, *n* = 136) = 4.739, *p* = .857, comparative fit index = 1.00, Tucker–Lewis index = 1.02, RMSEA < 0.001, 95% CI [0.000, 0.053], SRMR = 0.018, and accounts for 94% of the variance in single-word reading ability.

## General Discussion

We found moderate to strong correlations between speechreading and phonological awareness in deaf children and also in hearing children, despite the fact they have full access to auditory speech information. In addition, our measures of phonological awareness (which were different for the deaf and hearing groups) show moderate-to-strong correlations with single-word reading ability in both groups. This result is expected for the hearing children (see Melby-Lervåg et al., 2012), but evidence concerning the size of such a correlation in deaf populations is more mixed (see Mayberry et al., 2011). We also found that the relationship between speechreading and single-word reading ability in both young deaf and hearing children was fully mediated by phonological awareness. Although the direction of any causal relationships cannot be determined from the concurrent data presented here, our findings are consistent with the theory outlined in the introduction that visual information derived from speechreading contributes to the development of phonological representations in both deaf and hearing children and that phonological representations, in turn, are critical for performance on both phonological awareness tasks and for single-word reading development. Such a theory sees phonological awareness as a proxy for the integrity of phonological representations and postulates unidirectional causal effects (speechreading → phonological representations → single-word reading). However, it is clear that other causal relationships are possible. For example, it may be that the quality of phonological representations has a causal effect on both speechreading and single-word reading in both deaf and hearing children rather than mediating the relationship between them. That is, good phonological awareness might lead to the development of not only good single-word reading but also good speechreading skills. The plausibility of these different possible causal interpretations in the two groups is further explored below.

It is worth highlighting that, in the current sample of deaf children, phonological awareness was strongly related to single-word reading ability (*r* = .61 for onset matching with YARC Single-Word Reading subtest). This supports numerous previous studies that have reported a correlation between phonological awareness and reading development in deaf children (Campbell & Wright, 1988; Dyer et al., 2003; Harris & Beech, 1998; Herman et al., 2019). Factors such as the age of the children tested and the measures used are likely to influence the strength of this relationship, and these factors may explain the inconsistencies between previous studies (see Mayberry et al., 2011). The proportion of variance in single-word reading scores explained by the models in the current study was notably high for both deaf (*R* ^2^ = .75) and hearing children (*R* ^2^ = .94), which may in part reflect our use of latent variable models, which control for measurement error (see Hjetland et al., 2019).

Study 1 replicated the positive relationship between speechreading and single-word reading in deaf children (e.g., Harris et al., 2017a; Kyle et al., 2016; Rodríguez-Ortiz et al., 2017). Importantly, our data also showed that, in deaf children, the relationship between speechreading and single-word reading was fully mediated by phonological awareness. As noted earlier, this is consistent with the suggestion that speechreading may facilitate the development of better specified phonological representations, which in turn facilitate learning the mappings between written letters and those representations.

In Study 2, we found a similar pattern in hearing children to that in the deaf children in Study 1. The role of visual speech perception in hearing children's reading is relatively underexplored. Our previous studies have suggested a correlation between speechreading and single-word reading in hearing children (Kyle et al., 2016; Kyle & Harris, 2011). The current study furthers our understanding of this relationship. Previous studies have suggested that hearing infants are sensitive to visual speech information (Kuhl & Meltzoff, 1982; Patterson & Werker, 1999, 2003) and that it facilitates speech segmentation and learning phonetic boundaries (Jerger et al., 2018; Teinonen et al., 2008; Weikum et al., 2007). Study 2 suggests that visual speech perception may play a similar role in supporting the development of phonological representations in hearing, as in deaf, children. The correlations between performance on the speechreading and phonological awareness tasks were strikingly similar between the two studies (deaf children: onset matching *r* = .451, rime matching *r* = .587; hearing children: phoneme deletion *r* = .471). However, it is notable that the relationship between the latent variables representing speechreading and phonological awareness was rather weaker in the hearing sample (β = 0.445) than in the deaf sample (β = 0.748). This difference in the strength of relationship may have a number of explanations, including the different tasks used (alliteration and rhyme matching for the deaf children, phoneme deletion for the hearing children) or that auditory speech information is more critical as a foundation for phonological development in hearing children. Despite a slightly weaker relationship, the results for hearing children support and extend previous studies that show an association between speechreading and phonological awareness in hearing children (Heikkilä et al., 2017, 2018; Kyle & Harris, 2011). These studies highlight the multimodal nature of speech processing and phonological development in both hearing and deaf populations.

In line with the literature reviewed in the introduction (e.g., Kyle, 2015), our favored interpretation of the pattern of results in both deaf and hearing children is that visual speech information contributes to the development of phonological representations, which in turn are crucial for the development of both phonological awareness and single-word reading skills. In this view, phonological representations, in both hearing and deaf populations, are inherently multimodal involving auditory, visual, and articulatory information. However, as already noted, these are concurrent data, and other causal relationships are possible. It is also possible that different relationships would be evident between these factors at different developmental stages. For example, we have previously shown that, in deaf adults, the relationship between speechreading and reading remained (*r* = .49) when phonological awareness was controlled for (Mohammed et al., 2006). This suggests that, although speechreading relates to phonological awareness skills, there may be other factors contributing to the relationship between speechreading and reading in deaf adults.

As suggested above, it may be that phonological representations have a causal effect on both speechreading and single-word reading rather than mediating the relationship between them. That is, good phonological representations might lead to the development of good single-word reading and speechreading skills. Hearing children are likely to establish phonological representations primarily through auditory speech perception and may then use these representations to aid speechreading. However, many studies indicate that visual speech information can influence speech perception in both hearing adults and infants (e.g., Sumby & Pollack, 1954). Therefore, for hearing children to be able to map from auditory speech representations to speechreading, they must have established multimodal phonological representations.

Although in hearing children it may be that phonological representations facilitate speechreading (phonological representations → speechreading) rather than the other way round (speechreading → phonological representations), such a reverse relationship seems much less likely in deaf children because visual information about speech gestures is a key source of information about the structure of spoken language for deaf children. Therefore, although deaf children may develop their phonological representations through reading to some extent, speechread information is likely to contribute to the development of phonological representations in deaf children (Kyle, 2015). Although it is possible that phonological awareness is a cause of both speechreading and single-word reading development, we believe it is more likely that visual speech perception contributes to establishing robust multimodal phonological representations in both deaf and hearing children.

### Limitations

One challenge in conducting research with small populations, such as severely to profoundly deaf children, is obtaining sufficient sample sizes. Our sample of 66 deaf children is relatively large in relation to typical studies with this population but is still relatively small for conducting structural equation modeling. Nevertheless, the model reported here is a relatively simple one and provided a good fit to the data. One limitation arising from the small sample size is that we could not explore how the model with deaf children might vary depending on the child's language background (spoken English only, mixture of speech and sign, BSL) or access to auditory speech through technology (hearing aids or cochlear implants). While the diversity in language backgrounds and use of technology of the current sample is representative of the population, care should be taken in applying this model to specific subgroups of deaf children without further study into how the model may vary. It should also be stressed that, although the relationship between speechreading and single-word reading is fully mediated by phonological awareness in the current models, this does not preclude the possibility that other factors, such as vocabulary, may play a mediating role in this relationship (Rucker et al., 2011). Language was not included as a latent variable in the current studies, as the aim was to address the specific question of whether phonological awareness skills mediate the relationship between speechreading and single-word reading, rather than to build a comprehensive model of reading ability in deaf and hearing children.

As the mediation models in the current studies are based on concurrent data, the direction of causality for the relationship between speechreading, phonological awareness, and single-word reading cannot be established. A strong test of the causal theory outlined here would require evidence from a training study, the prediction being that if speechreading can be trained effectively, it should produce effects that generalize to improvements in phonological awareness and single-word reading ability. Our recent training study (Pimperton et al., 2019) found that 12 weeks of speechreading training (10 min/day) with deaf children led to gains in speechreading, trained vocabulary, and speech output, which was used as a measure of the quality of their phonological representations. However, there was no effect on single-word reading or on explicit measures of phonological awareness (onset and rime matching) as would be predicted by the current mediation models. These null effects of training on single-word reading are difficult to interpret: Clearly, the absence of such effects does not mean that such effects cannot be obtained. Future training studies are necessary to establish whether longer training or targeting different levels of speech analysis may indeed lead to subsequent gains in single-word reading.

In summary, we have found a relatively strong relationship between speechreading and single-word reading, which is mediated by phonological awareness in both deaf and hearing children. This is consistent with the idea that the visual information about speech gestures conveyed by speechreading is important for the development of phonological representations, which in turn support reading development. However, further longitudinal and training studies (with both deaf and hearing children) are needed to clarify the causal relationships involved.
